# Heterogeneous nuclear ribonucleoprotein U drives a pro-regenerative astrocyte response

**DOI:** 10.4103/NRR.NRR-D-25-01031

**Published:** 2025-12-30

**Authors:** Ruijuan Zhang, Lili Quan, Rieko Muramatsu

**Affiliations:** Department of Molecular Pharmacology, National Institute of Neuroscience, National Center of Neurology and Psychiatry, Tokyo, Japan; Department of NCNP Brain Physiology and Pathology, Graduate School of Medical and Dental Sciences, Institute of Science Tokyo, Tokyo, Japan

**Figure 1:**
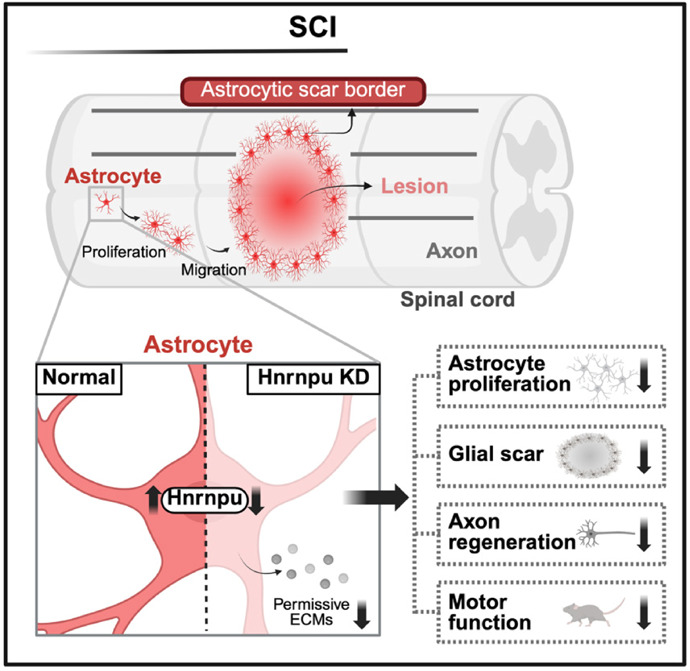
Schematic illustrating the role of astrocytic Hnrnpu in promoting astrocyte proliferation, scar formation, and functional recovery following SCI. Astrocytes are the primary component of the glial scar border, participating in scar formation through proliferation, migration, and reactivation. Hnrnpu knockdown in astrocytes leads to reduced astrocyte proliferation and scar formation, downregulation of permissive ECM molecules, as well as suppressed axon regeneration and motor function. Created with BioRender.com. ECM: Extracellular matrix; Hnrnpu: heterogeneous nuclear ribonucleoprotein U; KD: knockdown; SCI: spinal cord injury.

Traumatic spinal cord injury (SCI) is a devastating central nervous system (CNS) disorder characterized by significant neurological dysfunction and sensory loss, and effective therapies that prevent neuronal loss and functional recovery remain elusive. After SCI, lesions are surrounded by neuroprotective borders formed by newly proliferated reactive astrocytes. Astrocyte proliferation and activation mediate the formation and function of the glial scar and influence the balance between protection and inflammation. However, molecular mechanisms that regulate these essential astrocytic responses are still poorly understood. Our recent study highlights the DNA/RNA-binding protein, heterogeneous nuclear ribonucleoprotein U (Hnrnpu), as a potential endogenous regulator in astrocyte proliferation, migration, and subsequent scar formation (Quan et al., 2025). Notably, Hnrnpu appears to selectively enhance the expression of permissive extracellular matrix (ECM) molecules, thereby promoting axon regrowth and revealing a pro-regenerative astrocyte response. These findings deepen the understanding of the intrinsic mechanisms that control astrocyte response and provide a new regulatory mechanism involved in reactive astrocyte proliferation and resulting beneficial effects on CNS injury repair. Overall, this work demonstrates the essential function of glial pathology in response to CNS injury and points to a promising therapeutic approach for promoting axon regeneration through targeting astrocytic Hnrnpu to modulate glial scar formation (**Figure 1**).

**Rethinking the glial scar — beyond the barrier:** It is traditionally believed that the glial scar is a barrier hindering axon regeneration and effective functional repair after CNS injury (Tran et al., 2018). This view is grounded in the evidence that after CNS injury, the reactive astrocytes proliferate, migrate to the lesion, and secrete a dense ECM particularly rich in inhibitory molecules such as chondroitin sulfate proteoglycans (CSPGs), which form both structural and biochemical barriers, inducing the failure to restore the structure, composition, and function of spinal cord. This view has led to extensive efforts to aim at strategies such as scar ablation or astrocyte reactivity suppression for promoting repair. However, the supportive functions of astrocytic scar formation have been increasingly recognized in recent years. Glial scar also plays an important role in inhibiting inflammation, restricting lesion expansion, preserving tissue integrity, and providing a microenvironment beneficial to neuron repair (Bradbury and Burnside, 2019). Rather than being an obstacle, the glial scar is now widely accepted as a dynamic and multifunctional structure whose effect depends on the balance between promoting and inhibiting regenerative factors (Tran et al., 2018). In addition, the contrasting roles of CSPGs in inhibiting or supporting axon growth have been reported. While inhibitory CSPGs such as aggrecan, brevican, and neurocan are widely discussed, many CSPGs, including NG2 (CSPG4), have been found to play supportive functions in axon growth, stabilize the lesion site, and limit secondary damage and inflammation (Bradbury and Burnside, 2019). For CNS repair, the question has shifted from “How to eliminate the glial scar?” to “How to understand and fine-tune its function?” (Bradbury and Burnside, 2019). This shift requires a deeper understanding of molecular and cellular mechanisms that shape astrocyte phenotypes after injury.

**Astrocyte diversity and plasticity — the new frontier:** Astrocytes are the primary cellular component of glial scars, shaping the post-injury environment (Burda and Sofroniew, 2014). Reactive astrocytes populate the injury site, upregulate the expression of intermediate filament proteins such as glial fibrillary acidic protein, and form a barrier that restricts and protects the lesion area (Liddelow and Barres, 2017; Escartin et al., 2021). One of the most important recent developments is the recognition of astrocyte heterogeneity and plasticity. Astrocytes are now recognized for their high capacity to change their molecular, morphological, and functional profiles in response to injury and disease (Endo et al., 2022). Astrocytes have been shown to undergo dynamic changes, for example, changes in specific genes and proteins like vimentin and cytokines, accompanied by the morphology transition to hypertrophy or process retraction, leading to improved, worsened, or unchanged functional outcomes (Escartin et al., 2021). There are multiple subtypes of reactive astrocytes. Some are associated with inflammation and inhibition, while others are associated with neuroprotection and repair (Ngoc et al., 2025). What leads the direction of glial scar-forming astrocytes toward beneficial or harmful phenotypes?

While much attention has been paid to extrinsic factors such as inflammatory cytokines, growth factors, and extracellular signals in driving astrocyte reactivity and glial scar formation, the intrinsic cell regulation program, particularly at the transcriptomic level within astrocytes that governs their response to injury, has been less explored. Since astrocyte responses are partly driven by complex gene expression changes, clarifying these transcriptome mechanisms is essential for understanding the context-dependent role of astrocytes in injury repair (Escartin et al., 2021).

**Emergence of Hnrnpu — a new regulator of driving pro-regenerative astrocyte response:** Astrocyte proliferation is a hallmark of reactivity and crucial for glial scar formation and repair microenvironment shaping (Burda and Sofroniew, 2014). Our study identified Hnrnpu as a novel intrinsic regulator of astrocyte proliferation. Through siRNA screening targeting genes enriched in Gene Ontology terms related to “cell proliferation” in pathological spinal cord astrocytes, we found that Hnrnpu exerts the most significant inhibitory effect on primary astrocyte proliferation *in vitro*. Hnrnpu, a member of the heterogeneous nuclear ribonucleoprotein family, is a nuclear DNA- and RNA-binding protein known for its roles in transcription, alternative splicing, and cellular proliferation (Geuens et al., 2016). Its importance in neural development and disease has been recognized, with genetic mutations linked to intellectual disability and epilepsy. Although Hnrnpu is widely expressed in various cell types, its function in astrocytes remains to be fully explored.

The role of Hnrnpu in regulating astrocyte proliferation and migration *in vitro* was confirmed in our study. We further investigated whether Hnrnpu affects astrocyte proliferation *in vivo* using an SCI mouse model. After injury, Hnrnpu expression was upregulated in reactive astrocytes, exhibiting a temporal pattern similar to that of proliferating astrocytes. Using an astrocyte-specific adeno-associated virus vector to manipulate Hnrnpu expression, we found that inhibition of Hnrnpu led to decreased astrocyte proliferation, impaired glial scar formation, expansion of the lesion core, reduced axon regeneration around the injury site, and inhibited motor function recovery. These results suggest that Hnrnpu is an important endogenous factor driving the injury response of astrocytes, proliferation, and protective scar formation, thereby promoting tissue repair and functional recovery. As astrocytic scar formation is mainly formed by proliferating astrocytes around the lesion site, we primarily focus on the function of Hnrnpu in this region. Since Hnrnpu also affects cell migration, it would be intriguing to further clarify its effect on spatially distinct astrocyte populations and to better understand the precise role of Hnrnpu in astrocyte heterogeneity after injury. We also confirmed whether these findings are conserved in human astrocytes. Inhibition of Hnrnpu in astrocytes isolated from the human brain cortex resulted in suppressed astrocyte proliferation, as measured by BrdU incorporation and immunostaining for proliferating astrocytes, suggesting that Hnrnpu is also required for human astrocyte proliferation. In summary, our results demonstrate that Hnrnpu is a key intrinsic regulator of astrocyte proliferation and the following scar formation, with conserved roles in both mouse and human astrocytes, highlighting its potential as a therapeutic target for CNS repair.

Regarding the mechanism of Hnrnpu in astrocytes, we found that Hnrnpu inhibition selectively suppresses pro-regenerative factors within scars, such as laminins (LAMA5 and LAMB2) and permissive proteoglycan CSPG4, at both the transcriptional and protein levels. Our transcriptomic analyses further indicated that Hnrnpu may regulate astrocyte proliferation through pathways related to MYC targets, E2F targets, and mTORC1 signaling. However, further studies are needed to clarify the molecular mechanisms underlying Hnrnpu-regulated astrocyte reactivity.

**Targeting astrocytes to promote neural regeneration:** Astrocytes, the most abundant glial cells in the CNS, play a comprehensive role in responding to CNS injuries. In particular, their response to injury involves the formation of a glial scar, the dual functions of which are now widely recognized. The identification of Hnrnpu as an intrinsic activator that promotes astrocyte proliferation and beneficial scarring offers new therapeutic angle: rather than broadly inhibiting scar formation, targeting Hnrnpu could specifically induce the pro-regenerative aspects of astrocytic scar formation, thereby minimizing inhibitory effects and facilitating repair. Theoretically, enhancing Hnrnpu activity in astrocytes through gene therapy or small-molecule agonists could promote protective scarring and create a more favorable environment for recovery. Additionally, Hnrnpu inhibition does not affect the inhibitory CSPGs expression, such as aggrecan, brevican, and neurocan, or inflammatory responses *in vivo*, suggesting minimal potential side effects. This study opens several avenues for future investigation. A primary goal is to explain the complete downstream transcriptional network of Hnrnpu to identify its direct targets and regulatory motifs. Furthermore, elucidating the upstream signals that induce Hnrnpu expression after injury may provide additional therapeutic targets. For example, Toll-like receptor signaling has been demonstrated to upregulate Hnrnpu protein expression and stimulate its translocation from the nucleus to the cytoplasm, thereby inducing proinflammatory cytokine production (Zhao et al., 2012). Targeting these pathways could potentially modulate the inflammatory response and improve functional recovery after CNS injury.

Astrocytes should not be considered as “good” or “bad” but as a diverse and highly plastic population dynamically regulated by both intrinsic and extrinsic molecules. Astrocytes comprise several subtypes with distinct molecular profiles and functional roles in CNS injury and diseases (Escartin et al., 2021). We have shown that the inhibition of Hnrnpu may impact motor function recovery and neural regeneration in mice, suggesting that Hnrnpu activation may drive astrocytes to a beneficial phenotype for repair. Understanding how Hnrnpu functions with different astrocyte subtype may help to refine our understanding of repair involving astrocytes. The current study demonstrates that Hnrnpu might primarily target the proliferative astrocyte subpopulation. Specifically, Hnrnpu inhibition reduces the number of BrdU^+^Sox9^+^EGFP^+^ cells (proliferating astrocytes) *in vivo*. No changes are observed in the expression of reactive astrocyte marker proteins around the lesion site 7 days post-injury, suggesting that Hnrnpu may not be uniformly distributed across the entire astrocyte population but is likely restricted to proliferating astrocytes surrounding the lesion core. Further studies would be needed to determine whether the effect of Hnrnpu extends to other astrocyte subpopulations or disease models. Moreover, an emerging study has indicated that astrocyte phenotype changes after injury exhibit temporality and reversibility, depending on the lesion environment and molecular effect (Escartin et al., 2021). This evidence suggests a critical time window after injury may exist to maximize Hnrnpu-driving repair and sustain Hnrnpu regulatory requirements due to the potentially reversible nature of effective injury repair. Given that there are multiple cellular components involved in the glial scar, including astrocytes, microglia, oligodendrocytes, and immune cells, they interact with each other through cytokine- and chemokine-mediated cellular interactions. For example, resident microglia express receptors for cytokines and chemokines released by astrocytes. In turn, astrocytes also express receptors for the inflammatory mediator released by immune cells (Bradbury and Burnside, 2019). A better understanding of possible interactions between reactive astrocytes driven by Hnrnpu activation and other cell types may help to build a complete view of the glial scar. Finally, while the current study focuses on SCI, similar astrocyte-driven processes are at play in traumatic brain injury, stroke, and even chronic neurodegenerative conditions. It will be important to investigate whether Hnrnpu plays analogous roles in these contexts, potentially establishing it as a universal regulator of astrocyte-mediated repair.

In conclusion, our study identified Hnrnpu as an intrinsic regulator of pro-regenerative astrogliosis, providing an understanding of astrocytic scar formation in CNS repair. This advanced step encourages future therapy strategies moving from eliminating glial scars to enhancing their beneficial roles, presenting a new direction for treating CNS injuries by taking advantage of astrocyte plasticity.

*This work was supported by a Grant-in-Aid of AMED under Grant Numbers (JP22gm1510009) to RM.*
